# Individualized Estimation of Baseline Retinal Nerve Fiber Layer Thickness Using Conditional Variational Autoencoder

**DOI:** 10.1016/j.xops.2025.100849

**Published:** 2025-06-09

**Authors:** Ou Tan, Keke Liu, Aiyin Chen, Dongseok Choi, Jonathan C.H. Chan, Bonnie N.K. Choy, Kendrick C. Shih, Jasper K.W. Wong, Alex L.K. Ng, Janice J.C. Cheung, Michael Y. Ni, Jimmy S.M. Lai, Gabriel M. Leung, Ian Y.H. Wong, David Huang

**Affiliations:** 1 Casey Eye Institute, Oregon Health & Science University, Portland, Oregon; 2 Department of Ophthalmology, Duke University School of Medicine, Durham, North Carolina; 3 Department of Ophthalmology, LKS Faculty of Medicine, The University of Hong Kong, Hong Kong, Hong Kong Special Administrative Region, China; 4 School of Public Health, LKS Faculty of Medicine, The University of Hong Kong, Hong Kong, Hong Kong Special Administrative Region, China; 5 The State Key Laboratory of Brain and Cognitive Sciences, The University of Hong Kong, Hong Kong, Hong Kong Special Administrative Region, China; 6 Healthy High Density Cities Lab, HKUrbanLab, The University of Hong Kong, Hong Kong, Hong Kong Special Administrative Region, China; 7 Department of Ophthalmology, Hong Kong Sanatorium & Hospital, Hong Kong, Hong Kong Special Administrative Region, China

**Keywords:** Conditional variational autoencoder, Glaucoma, Nerve fiber layer thickness, OCT

## Abstract

**Purpose:**

Use generative deep learning (DL) models to estimate baseline reference nerve fiber layer thickness (NFLT) profiles, taking into account individual ocular characteristics.

**Design:**

A cross-sectional study.

**Participants:**

Six hundred eighty-six individuals from the Hong Kong FAMILY cohort and 75 individuals from the Casey Eye Institute (CEI) cohort.

**Methods:**

Healthy eyes were selected from the Hong Kong FAMILY and CEI cohorts. Circumpapillary NFLT profiles and vascular patterns were measured by a spectral-domain OCT. Generative DL models were trained using the FAMILY data to reconstruct the individualized baseline NFLT, a customized normal reference based on each eye’s own vascular pattern, axial length (AL), spherical equivalent (SE) refractive error, disc size, and demographic information. Two DL models were developed. The MAG model used actual AL and SE, while the REG model estimated AL and SE using vascular patterns as input. For comparison, a multiple linear regression (MLR) was trained to estimate baseline NFLT using AL and demographic information. Fivefold cross-validation was used to assess performance.

**Main Outcome Measures:**

The prediction error: root-mean-square of the difference between the actual NFLT profile and the predicted individualized baseline.

**Results:**

A total of 1152 healthy eyes from 686 participants in the Hong Kong Family cohort were divided into 4 subgroups: high myopia (SE <−6 diopters [D]), low myopia (SE = −6 D ∼ −1 D), emmetropia (SE = −1D∼1D), and hyperopia (SE >1D). Compared with the population means, both DL models significantly reduced the prediction error for overall and quadrant NFLT and decreased the false-positive rate of identifying abnormal NFLT thinning in both myopia groups (from 13.0%-27.0% to 6.3%∼9.4%). Both DL models significantly reduced prediction error for the NFLT profiles compared with both the population mean and the MLR-adjusted NFLT. The reductions in prediction errors for NFLT profile and overall NFLT value were independently validated using the CEI data.

**Conclusions:**

Generative DL models (a type of artificial intelligence) can construct individualized NFLT baseline profiles using the vascular pattern derived from the same OCT scans. The individualized baseline reduced the prediction error of the NFLT profile in healthy eyes and may improve the accuracy of identifying abnormal NFLT thinning, especially in myopic eyes.

**Financial Disclosure(s):**

Proprietary or commercial disclosure may be found in the Footnotes and Disclosures at the end of this article.

Glaucoma is associated with the progressive loss of retinal nerve fiber layer thickness (NFLT).^1^, ^2^, ^3^ OCT is commonly used to detect and monitor nerve fiber layer thinning.^4^^,^^5^ Detection of pathological thinning is based on the premise that the NFLT measured in an individual person can be compared to a theoretical baseline value before disease onset. In conventional practice (i.e., the glaucoma diagnostic packages offered by OCT manufacturers), NFLT distribution in a healthy population is usually used as a generic baseline. Usually, a normative database with hundreds of healthy eyes would be collected for a specific OCT model in the US Food and Drug Administration clearance process. Nerve fiber layer thickness variation associated with demographic (age, race, and sex) factors is accounted for by regression or matching with the patient to be tested. However, the conventional approach neglects significant variations in the NFLT associated with the vascular pattern and transverse optical magnification of the individual eye. Neglecting these individual variations can lead to false-positive diagnoses or false-negative misdiagnoses of glaucoma and other optic neuropathies.

A well-known problem of the conventional approach is the false-positive diagnosis of glaucoma in highly myopic eyes, which have thinner measured NFLT globally (and in most sectors) due to higher axial length (AL) and lower transverse optical magnification.^6^, ^7^, ^8^, ^9^, ^10^, ^11^ The lower magnification causes the OCT scan pattern to span a larger area around the optic nerve head (ONH), and it is well known that the NFLT decreases with greater distance from the ONH in a roughly reciprocal fashion.^8^ This problem is so common that it has earned the name “red disease” because values below the 1 percentile cutoff of the healthy population are typically printed in red on OCT displays.^12^

It is also known that the thickness peaks in the NFLT profile are at the superior and inferior arcuate bundles that generally collocate with the major arcade vessels.^13^ Thus, the peripapillary vascular pattern measured on the OCT can be used to predict the NFLT peak location. The vascular pattern also contains information on the papillomacular axis, which is also known to influence the NFLT pattern.^14^, ^15^, ^16^, ^17^, ^18^ Disc size is another measurable individual characteristic that is known to influence NFLT.^8^^,^^19^

We define the individualized baseline as the customized normal reference that accounts for individual eye characteristics such as the vascular pattern and the transverse optical magnification, which can be represented by its determinants: the AL and spherical equivalent (SE) refractive error. We hypothesize that an individualized baseline NFLT profile can serve as a better reference for detecting pathological thinning associated with glaucoma and other optic neuropathies. We further hypothesize that it is feasible to obtain an individualized baseline using the map of major retinal blood vessels that can be extracted from the same OCT scan used to produce the NFLT map, plus AL, SE, and demographic information. To generate the individualized baseline, we chose a deep learning (DL) generative model, a conditional variational autoencoder (CVAE).^20^, ^21^, ^22^, ^23^

Recent studies have successfully used DL models to predict clinical features, such as gender, race, and AL.^20^^,^^24^, ^25^, ^26^, ^27^, ^28^ This encourages us to use DL to analyze the vascular pattern to generate information on AL and SE. We chose to use a convolutional neural network for regression analysis (rCNN)^29^ to estimate individual features, such as AL and SE.

We developed 3 DL models based on how the AL/SE was used: no AL/SE; measured AL/SE; and AL/SE estimated by an rCNN. We compare their performance to a regression model. The performances for these various baseline references are compared by the prediction error, the root-mean-square of the difference between the true NFLT parameters and the generated baselines. We also calculated the false positive rate (FPR) of detecting significant NFLT loss, stratified by myopic refractive error, to assess the effectiveness of reducing the “red disease” problem. The difference between the reference NFLT with the 5 percentile cutoffs is used as a preliminary estimation of potential gain in glaucoma diagnostic sensitivity.

## Methods

### Participants

#### Hong Kong Dataset

This cross-sectional study included participants from the Hong Kong FAMILY Cohort, a large territory-wide random sample of occupants from several Hong Kong districts. The Institutional Review Board of the University of Hong Kong approved the study, which adhered to the Declaration of Helsinki. Comprehensive details of the recruitment process and cohort characteristics were previously reported.^30^ In brief, all participants older than 18 years were invited to participate and provided consent before enrollment. All participants received a comprehensive ophthalmic examination, including visual acuity, subjective refraction, perimetry, keratometry, pachymetry, axial eye length, intraocular pressure (IOP), slit-lamp examination, and indirect ophthalmoscopy.^31^ The axial eye length was measured with an ocular biometer (AL-Scan, Nidek).

Only healthy eyes were included in this study, and either 1 or 2 eyes were included per participant. The exclusion criteria were the following: subjects with a history of glaucoma, abnormal test in frequency doubling technology perimetry or fundus examination (disc, macula, or vessels), elevated IOP (>21 mmHg), enlarged cup-to-disc ratio (>0.7), pseudophakia, or missing data.

#### Casey Eye Institute Dataset

This case-control study was performed at the Casey Eye Institute (CEI), Oregon Health & Science University. The research protocol was approved by the institutional review board at Oregon Health & Science University and adhered to the tenets of the Declaration of Helsinki. Written informed consent was obtained from each participant.

Participants were part of the “Functional and Structural Optical Coherence Tomography for Glaucoma” study.^32^ The inclusion criteria for normal control were (1) no history of glaucoma, retinal pathology, or current corticosteroid use; (2) no history of ocular hypertension as defined by IOP ≥22 mmHg; (3) normal Humphrey 24-2 visual field test; (4) normal ONH and nerve fiber layer (NFL) appearance on funduscopy; (5) symmetric ONH appearance between both eyes; and (6) central pachymetry >470 μm. The exclusion criteria were (1) best-corrected visual acuity less than 20/40; (2) previous intraocular surgery except for uncomplicated cataract extraction with posterior chamber intraocular lens implantation; (4) any diseases that may cause visual field loss or optic disc abnormalities; and (5) narrow anterior chamber angle by gonioscopy. Only 1 eye of each participant received OCT scanning and analysis. This CEI dataset was introduced to obtain independent validation of models trained on the Hong Kong dataset.

### OCT Measurements

**Figure 1:**
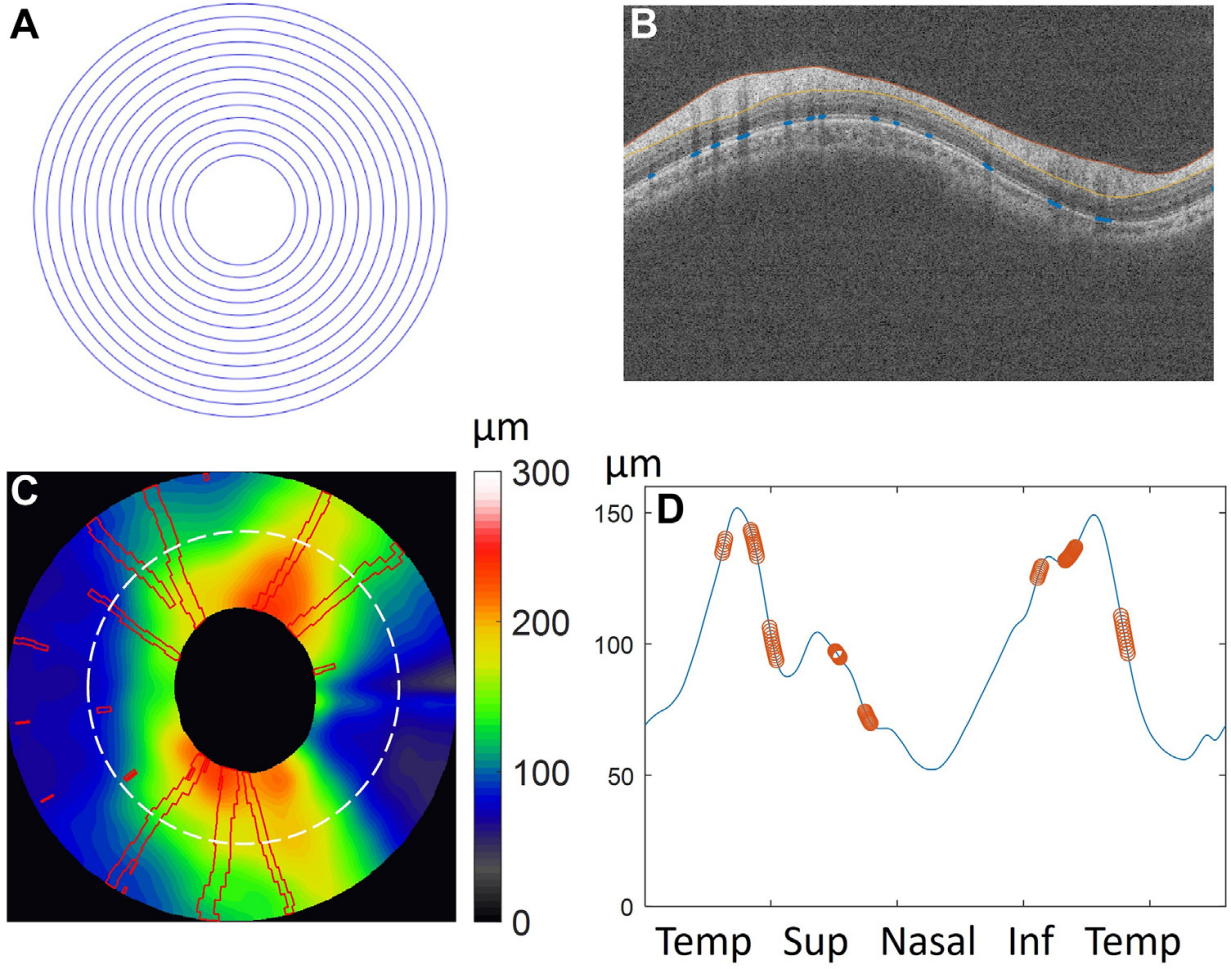
The NFLT map and vascular pattern. (**A**) Optic nerve head scan consists of circular scans (D = 1.3∼4.9 mm) covering the peripapillary area around the optic disc; (**B**) OCT B-scan with nerve fiber layer boundaries (solid lines) and vessel shadow detected (blue dots); (**C**) NFLT map and main vessel location map obtained from vessel shadow (red solid lines), the center black area masked the optic disc; (**D**) NFLT profile (blue line) and vascular pattern (red dots) were resampled on the recentered 3.4 mm circle (white dashed line in Figure 1C). The vascular pattern was used as a binary mask. D = diopter; NFLT = nerve fiber layer thickness.

The NFLT and disc size were obtained from an ONH scan using a commercially available spectral-domain OCT device (Avanti with AngioVue OCTA, Visionix/Optovue Inc). The ONH scan contains 13 rings covering a 4.9 mm peripapillary area around the optic disc (Figure 1A). Raw OCT images, boundary segmentation, signal strength defined by the signal strength index (SSI, 0-100), disc size defined by disc area in mm^2^, and the NFLT profile at diameter D = 3.4 mm were exported for data analysis in this study. An automated quality check algorithm was applied to OCT images to remove scans with poor SSI (SSI <35), retina cropping, or extremely low NFLT (NFLT value <4 times the population standard deviation in healthy eyes). Our previous study showed that SSI and disc size were also predictive factors of NFLT.^19^^,^^33^

The vessel location was detected using the shadow of large vessels in the inner retina on the retinal pigment epithelium (RPE) (Figure 1B). An *en face* map of vessel intensity in the RPE complex was reconstructed from the vessel shadow profile on the 13 rings of the ONH scan. Then we applied a level-set method^34^ to detect large vessels (Figure 1C).

The vascular pattern, NFLT, and boundary elevation profiles on different circles around the disc center were resampled from maps. Primarily, the NFLT profile and the vascular pattern profile at diameter = 3.4 mm were used as input for the generative DL model. The inner limiting membrane (ILM) and RPE elevation maps were also reconstructed, similar to the NFLT map.

### Generative DL Models

A CVAE was used to encode and generate the NFLT profile. A second CVAE was used to encode and reconstruct the vascular pattern. The generation of NFLT by the first CVAE was conditioned on the encoded vascular pattern from the second CVAE. Both CVAEs were conditioned by factors associated with an individual person, eye, and scan: age, sex, AL, SE, disc size, and scan signal strength. We picked the variational autoencoder architecture to address the issue of nonregularized latent space in the autoencoder and provide generative capability to the entire space. Variational autoencoders blend DL with probabilistic reasoning. Like an autoencoder, they have an encoder and decoder, but instead of just copying data, they learn the underlying probability distribution. This involves approximating the true distribution and minimizing the difference between them using a simpler one.^35^ Conditional variational autoencoder is a special type of variational autoencoder that adds individual factors to control the NFLT generation.^21^ Most importantly, we connected 2 CVAEs by using the hidden space of the CVAE of the vascular pattern as a conditional input of the NFLT CVAE. The latent space of the vascular pattern CVAE provided vectors corresponding to an individual vascular pattern. Those vectors made the generated NFLT profile match the specific vascular pattern.

We designed 3 models, depending on how to use magnification factors. The details are included in the next sections.

#### Models in the Training Stage

**Figure 2:**
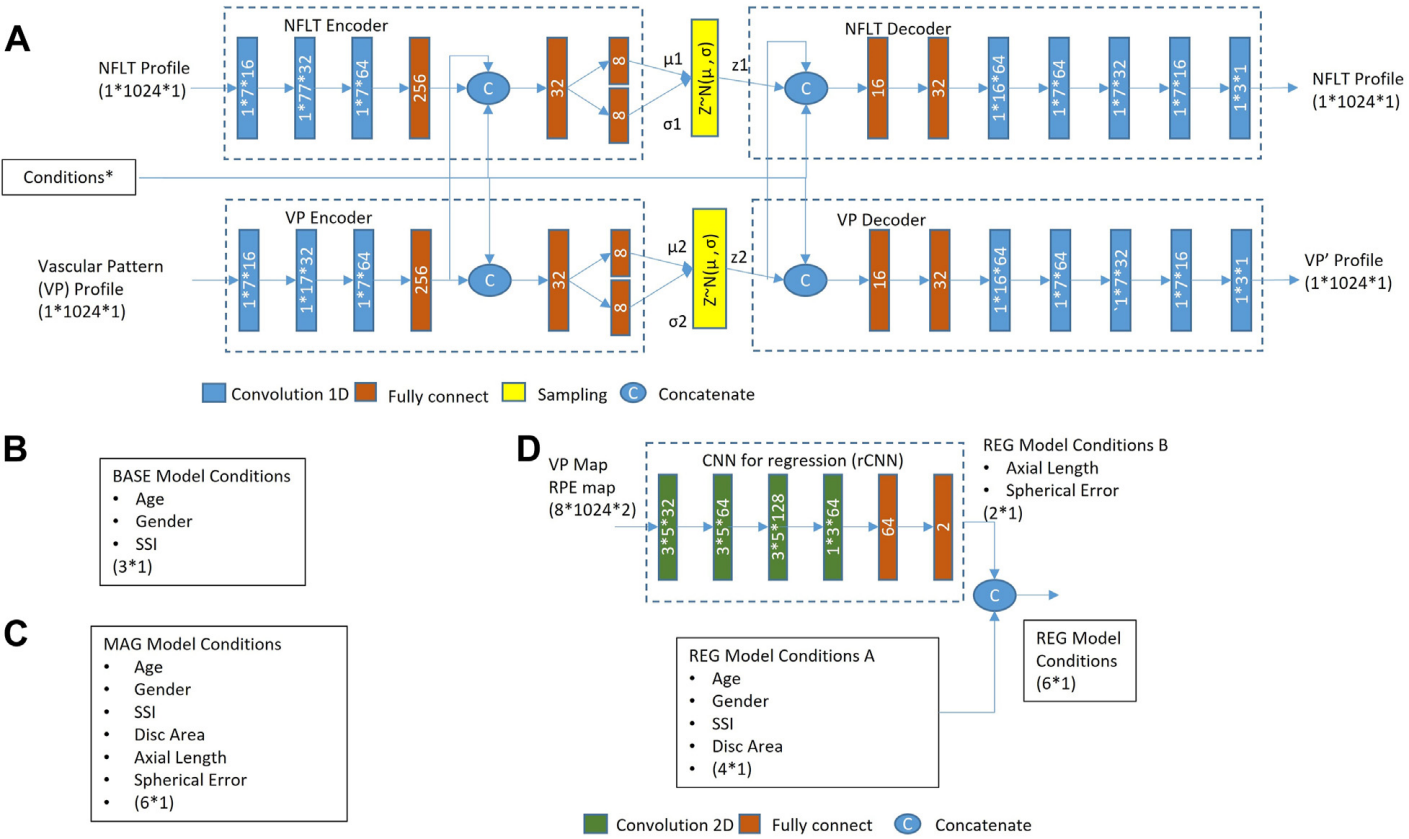
Training of the deep learning models. The retinal NFLT profile was used as a numeric array, and the VP profile was used as a binary array. (**A**) Overall structure of deep learning models in the training stage. It consists of a dual conditional variational autoencoder to reconstruct both the VP profile and the NFLT. Condition blocks were different for the 3 deep models: BASE, MAG, and REG. The details of the conditions were shown separately in Figure 2**B**–**D**. Note that the VP encoder’s output was also used as a condition for the NFLT encoder and decoders. (**B**) Conditions of the BASE model include only age, gender, and SSI. (**C**) Conditions of the MAG model include conditions listed in the BASE model, plus disc area, measured axial length (AL), and SE error; (**D**) Conditions of the REG model are similar to the MAG model. However, AL and SE were predicted by an rCNN. The rCNN used an RPE elevation map and vascular pattern map, and a binary mask of vessels was shown in Figure 1C. BASE = deep learning models using only demographic information; CNN = convolutional neural network; MAG = BASE model plus magnification information; MLR = multiple linear regression model; NFLT = nerve fiber layer thickness; NFLT’ = estimated nerve fiber layer thickness; rCNN = convolutional neural network for regression; REG = BASE model with magnification estimated with a regression convolutional neural network; RPE = retinal pigment epithelium; SE = spherical equivalent; SSI = signal strength index; VP = vascular pattern; VP’ = estimated vascular pattern; Z1 and Z2 = latent variable Z ∼ N (μ,σ): sampling in a latent space following a normal distribution of mean μ and standard deviation σ; 1D = 1-dimensional; 2D = 2-dimensional.

We first proposed a deep learning models using only demographic information (BASE) model, which has 2 parallel CVAEs (Figure 2A). Each CVAE had 3 parts: encoder, decoder, and sampling block. In the encoder, firstly, we used a convolutional neural network with several convolution blocks to convert profiles from 1 dimension in space into vectors in a higher dimension and reduce the transverse size to 1. Then, we used a concatenate layer that combined the output of convolutional neural network with conditions. A fully connected network converted the concatenated vector into vectors representing the μ and σ in the latent space. The sampling blocks created random variables z∼ N (μ, σ). In the decoder, the random variables were concatenated with conditions again. Then, a fully connected network, followed by several transverse convolution blocks, reconstructed the profile. Conditions were different between the 2 CVAEs. The vascular pattern CVAE used demographic information, such as age and gender, as a condition. The NFLT CVAE used the demographic information, plus the output from the vascular pattern CVAE, as the conditions. In the NFLT encoder, the output of the convolutional neural network of the vascular pattern CVAE was used as a condition for the vascular pattern. In the NFLT decoder, the output of the sample layer of the vascular pattern CVAE was used as a condition for the vascular pattern.

We then proposed a BASE model plus magnification information (MAG) model (Figure 2B). The structure was identical to the BASE model, except the conditions were updated to include magnification-related information, such as AL, SE, disc area, and SSI, besides the demographic information.

As AL and SE may not be available in commercial OCT, we also proposed a BASE model with magnification estimated with a regression convolutional neural network (REG) model, which used a predicted AL and SE based on OCT data. The magnification information had been estimated from the vascular pattern using disc photography in the literature.^27^^,^^36^^,^^37^ We used an rCNN to predict the AL and SE using information from OCT and demographic information (Figure 2C). The rCNN includes convolution blocks and fully connected networks. The input of rCNN included the vascular pattern map (Figure 1C) and the RPE elevation map. We did not use the ILM elevation map because glaucoma would change the characteristics of the ILM surface.^38^ Due to the limitation of the scan pattern, we used the available maps around the disc center (diameter = 2∼4 mm). The rCNN was trained using the actual AL and SE data. Once trained, the AL and SE outputs of the rCNN were used to condition the CVAEs in the MAG model.

#### Loss Functions

The training is based on the minimization of the loss function. The loss function of a CVAE is a summation of reconstruction loss and regularization loss. The reconstruction loss included the mean square error (MSE, for continuous variables) or binary cross entropy (BCE, for binary variables) between the input and the estimated profile, while the regularization loss included Kullback–Leibler (KL) divergence between the distributions represented by the latent vector and a standard Gaussian distribution. The loss function of the rCNN is a summation of MSE of all outputs. Therefore, the loss function of the REG model was the summation of the loss functions of 2 CVAEs and 1 rCNN.

$$Loss=MSE_{NFLT}+BCE_{VP}+KL_{VP}+{KL}_{NFL}+MSE_{AL}+MSE_{SE}$$

Here, NFLT was for CVAE for the NFLT profile, vascular pattern was for CVAE for the vascular pattern profile, and AL and SE were for the rCNN.

The loss function of the BASE and MAG models is similar to the previous formula but removes the last 2 MSE terms corresponding to the loss function of rCNN.

$$Loss=MSE_{NFLT}+BCE_{VP}+KL_{VP}+{KL}_{NFL}$$

#### Models in the Generating Stage

**Figure 3:**
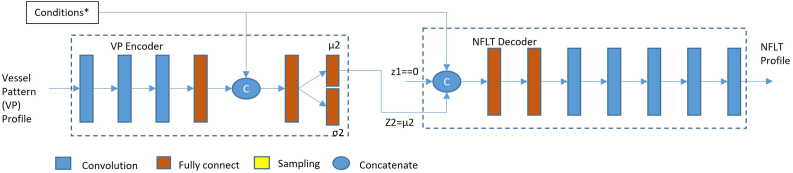
Generating the individualized baseline using deep learning models. (**A**) Overall structure of deep learning models in the generating stage. It is much simpler compared to the training stage. Only the VP encoder and NFLT decoder were used. The sampling layer is also removed to get the average NFLT, giving individual conditions. Therefore, the input of the NFLT decoder is the combination of Z1 = 0, Z2 = μ2, and other conditions. Condition blocks were different for the 3 deep models: BASE, MAG, and REG. The details of the conditions were shown in Figure 2. BASE = deep learning models using only demographic information; MAG = BASE model plus magnification information; MLR = multiple linear regression model; NFLT = nerve fiber layer thickness; REG = BASE model with magnification estimated with a regression convolutional neural network; VP = vascular pattern; Z1 and Z2 = latent variable.

In the generating stage, only the vascular pattern CVAE encoder and the NFLT CVAE decoder were used (Figure 3A–C). To generate the baseline NFLT value of the individual eyes, we set the input of the NFL-CVAE decoder (Z1) to 0. If we sampled the Z1 from N (0, 1), it allowed the generalization of a group of random vectors. If we combined them with a set of conditions, the NFLT decoder would generate a group of NFLT profiles corresponding to the same set of conditions. The variation in NFLT profiles will not be due to glaucoma damage, as the module is only trained on healthy eyes. We then might average them to get a normal reference for this given condition set. However, the average can also be approximated by using μ1 = 0 and σ1 = 0, which only needs to run the module once, therefore significantly reducing the calculation cost. So, we forced Z1 = 0 to generate the normal reference.

Similarly, we use the output μ2 vector from the vascular pattern encoder directly as Z2, indicating the vascular pattern part in the NFLT decoder's conditions. Note that μ was calculated, different from 0, and represented the specific vascular pattern. Using the earlier 2 simplifications, we generate a normal reference matching the individual vascular pattern and other conditions.

In the generating stage, the BASE model included demographic information and the μ vector form vascular pattern encoder; the MAG model included all conditions in the BASE model plus AL, SE, and disc area; and the REG model used similar conditions as the MAG model but replaced the AL and SE by the predicted value from the rCNN.

#### Normalization of Input

Glaucoma causes attenuation of retinal vascular caliber, which may affect the generation of individualized NFLT baseline reference and reduce the sensitivity of detecting disease. To avoid this potential pitfall, we normalized the vessel size of all eyes. The idea was to resize each vessel proportionally and keep a constant ratio of pixel numbers between vessels and nonvessels.

We normalized all inputs according to their mean and standard deviation to reduce the scale difference among features. The normalization accelerated and stabilized the learning process and avoided the problem of exploding gradients in the regression network.^39^

When both the left and right eyes were used for the same participant, the learning weight of each eye was halved in the training stage to equalize each participant's weight.

#### Training Algorithm and Parameter

In the training, we chose the Adam optimization algorithm (decay = 0.001) to update network parameters. Dropout (0.5) and L2 (0.02) regularization were used to reduce overfitting. The initial learning rate was set to 0.001.

### Multiple Linear Regression

The normative reference for NFLT can be improved by accounting for the effect of demographics, scan quality, and magnification. To assess the performance of this approach, we used multiple linear regression (MLR) with a mixed-effect model. Based on previous studies,^19^ we identified age, sex, and SSI and AL as the optimal combination of independent variables in the model. A broken stick (or segmented) regression model related NFLT to AL. With the breakpoints set at emmetropia (SE = 0 diopters [D]), the broken stick model divided healthy eyes into 2 segments: the hyperopia segment (SE >0) and the myopia segment (SE <0). The MLR was estimated for the overall average, sectoral averages, and each point on the NFL profiles. The normal reference is constructed by averaging all eyes after adjusting NFLT to a reference age/sex/SSI/AL based on the MLR. When we computed the prediction error or checked the abnormality of a testing eye, the NFLT was adjusted using the same scheme.

### Statistical Analysis

In total, 5 models of normal reference were compared in this study: (1) population average without any adjustment (average); (2) adjusted based on the MLR; (3) generated by the DL model without magnification information (BASE); (4) generated by the DL model with magnification information, using true AL and SE directly (MAG); and (5) generated by the DL model with AL and SE predicted by rCNN (REG).

Based on the Hong Kong dataset, fivefold cross-validation was used to test the performance of the fully trained models. For each fold, we used 80% of the eyes to train the DL/MLR models and the remaining 20% of the eyes to test the performance of the trained model. The final performance was pooled from all folds. In the training of DL models, 10% of the training set was reserved for internal validation (not to be confused with the fivefold validation) to avoid overfitting.

The prediction error was estimated as the root-mean-square of the difference between the true value and the predicted value using a mixed-effect model. The FPRs were compared among models using a generalized linear mixed-effect model equivalent to the McNemar test.^40^ All analyses were done in MATLAB R2021b with the Statistics Toolbox and Deep Learning Toolbox. Mixed-effect models were used to address between-eye correlation when applicable.

The models trained on the Hong Kong dataset were also applied to the CEI dataset. The prediction errors between the predicted normal reference and the tested eyes were estimated for the overall, quadrant, and profiles of NFL thickness. To compensate for the difference in NFLT due to race in 2 datasets, mainly East Asians in the Hong Kong dataset and multiple races in the CEI dataset, we proportionally adjusted the individualized baseline according to the ratio of the population average of NFLT profile between the emmetropia eyes in the 2 datasets. 41, 42, 43, 44, 45 To compare the prediction error among models, a mixed-effect model was fitted to each data set, followed by Dunnett (post hoc) test.

## Results

### Characteristics of the Study Participants

A total of 1152 healthy eyes from 686 participants with valid age, gender, AL, SE, and NFLT profiles were selected from the Hong Kong dataset (Table 1). Eyes were divided into 4 subgroups: 106 high myopia (SE <−6 D), 509 low myopia (−6 to −1 D), 401 emmetropia (−1 D to 1 D), and 136 hyperopia (>1 D). As expected, myopic eyes had longer AL, smaller disc size, and thinner NFL. Myopic eyes were also younger.

**Table 1:** Characteristics of the Study Subjects from 2 Cohorts

Variables	Hong Kong				CEI
	Hyperopia	Emmetropia	Low Myopia	High Myopia	
Participants (n)	92	233	299	62	75
Eyes (n)	136	401	509	106	75
Age (yrs)	59.2 ± 11.2	49.9 ± 13.6	41.9 ± 13.8	41.1 ± 11.5	58.6 ± 11.3
Female (%)	52.2	61.8	54.4	56.6	52.0
Spherical equivalent (D)	2.0 ± 1.5	0.1 ± 0.5	−3.1 ± 1.4	−7.8 ± 1.6	−1.8 ± 3.5
Axial length (mm)	23.3 ± 1.2	23.6 ± 1.0	25.1 ± 0.9	26.6 ± 1.0	24.3 ± 1.5
Disc area (mm^2^)	2.2 ± 0.4	2.2 ± 0.4	1.9 ± 0.4	1.7 ± 0.4	1.9 ± 0.3
Average nerve fiber layer thickness (μm)					
Overall	102.3 ± 9.0	102.7 ± 8.3	99.0 ± 8.9	95.5 ± 8.7	98.3 ± 9.5
Temporal quadrant	78.1 ± 9.6	78.2 ± 9.0	79.2 ± 9.8	82.5 ± 10.6	74.1 ± 10.8
Superior quadrant	124.7 ± 13.9	126.9 ± 12.9	121.5 ± 14.5	116.4 ± 14.6	117.8 ± 14.6
Nasal quadrant	76.6 ± 10.1	75.9 ± 10.2	69.4 ± 10.4	64.9 ± 10.8	77.0 ± 10.3
Inferior quadrant	129.8 ± 12.5	129.9 ± 12.9	125.8 ± 13.7	118.2 ± 13.8	124.2 ± 14.3

CEI = Casey Eye Institute; D = diopters.

Values for continuous variables are means ± standard deviations. Disc area is not magnification adjusted.

A total of 75 normal eyes from 75 participants were selected from the CEI dataset (last column, Table 1). The CEI dataset is relatively older and has fewer female participants than the Hong Kong dataset. A wide range of myopic eyes were included in the CEI dataset (SE = −14.5∼5 D, AL = 21.7∼29.0 mm), but the average SE, AL, and disc size of the CEI dataset were between emmetropia and low myopia in the Hong Kong dataset.

### Training of Regression and DL Models

Based on the Hong Kong dataset, the association of NFLT with predictive factors varied between quadrants (Table 2). In the MLR with a broken stick model, NFLT was significantly associated with AL (*P* < 0.001) for overall average and superior, nasal, and inferior quadrants. Significant associations were also found for age and gender, with different quadrant distributions. Those slopes based on the MLR were used to adjust the NFLT in later analyses.

**Table 2:** Association between NFL Thickness and Predictive Factors (Hong Kong Family Cohort)

Slopes	Group	Overall Average NFL	Quadrant Average NFL			
			Temporal	Superior	Nasal	Inferior
Axial length (μm/mm)†	Myopia	−2.65∗	0.72	−4.05∗	−3.22∗	−4.04∗
	Hyperopia	−1.47∗	0.41	−2.00∗	−2.16∗	−2.11∗
Age (μm/yrs)		−0.14∗	−0.12∗	−0.23∗	0.00	−0.20∗
Female (μm)		0.89	2.67∗	−1.23	−0.69	2.79∗
SSI (μm)		0.04	−0.00	0.10	0.02	0.02

NFL = nerve fiber layer; SSI = signal strength index.

∗ *P* < 0.005, spherical equivalent refractive error. Accounting for multiple comparisons of 4 quadrant values plus the overall value, the Bonferroni correction was used to set the *P* value cutoff at 0.01 for statistical significance. Gender difference is calculated by female – male.

† Slopes against axial length were different between the myopia segment and the hyperopia segment using the broken stick model, which divided the healthy eyes into 2 segments with a breakpoint at the spherical equivalent refractive error = 0.

In the REG model, the correlation coefficient between the predicted value and the ground truth is 0.59 ± 0.05 (*P* < 0.001) for AL and 0.54 ± 0.05 (*P* < 0.001) for SE, based on fivefold cross-validation. The correlation is moderate and much smaller than the correlation in the training, which was usually >0.70. This indicated overfitting in the regression model. Experiments by choosing a larger dropout rate, larger L2, or shallower network reduced the overfitting in the training, but the correlation in the test dataset was in the same range.

The estimation of binary cross-entropy loss of the vascular pattern of 3 DL models was similar (0.372∼0.375). The MAG and REG models had smaller root MSEs of NFLT prediction (8.27 and 8.20 μm) than the BASE model (8.95 μm).

### Prediction Error between Actual NFLT and the Predicted Baseline Reference

A smaller NFLT prediction error in healthy eyes means that a tighter diagnostic threshold can be used at a given specificity level. This helps to detect glaucoma at an earlier stage when NFLT thinning is more subtle. We calculated the difference between the actual individual NFLT values and the predicted baseline reference for each of the 5 models. Then we compared the prediction error of the more advanced models to the simplest model (unadjusted population average), for which the prediction error was simply the population standard deviation in the test dataset.

Based on the Hong Kong dataset, the MAG and REG models had the smallest prediction error for the overall and quadrants (Table 3). They were significantly lower than the normal reference based on the population average. However, they were only significantly better than the MLR model in nasal (*P* ≤ 0.01), but borderline for others (*P* < 0.10). The BASE model did not significantly reduce the prediction error compared with the population average without adjustment.

**Table 3:** Prediction Error (μm) of Nerve Fiber Layer Thickness in Healthy Eyes in 2 Cohorts

Models	Overall	Quadrant Average			
		Temporal	Superior	Nasal	Inferior
Hong Kong					
Average	8.99 ± 0.58	9.63 ± 0.44	14.28 ± 1.04	11.08 ± 0.73	13.74 ± 0.35
MLR	8.32 ± 0.57∗	9.36 ± 0.30	13.35 ± 0.85†	10.31 ± 0.84†	12.60 ± 0.48∗
BASE	8.93 ± 0.59	8.97 ± 0.37†	13.76 ± 0.78	10.75 ± 0.88	13.61 ± 0.54
MAG	8.18 ± 0.51∗	8.99 ± 0.25†	13.15 ± 0.82∗	9.65 ± 0.70∗	12.91 ± 0.52†
REG	8.16 ± 0.47∗	9.04 ± 0.33†	13.07 ± 0.64∗	9.56 ± 0.64∗	12.98 ± 0.39†
Casey Eye Institute					
Average	9.63	10.82	14.42	10.58	14.27
MLR	8.67	10.01∗	13.13	10.13	13.17
BASE	9.10†	9.89†	13.61	11.42	13.63
MAG	8.41†	9.57	12.56	9.52	13.66
REG	8.72∗	9.98	13.23	10.46	13.90

BASE = deep learning model trained with conditions of vascular pattern and demographic information; MAG = BASE model with extra conditions of measured axial length and spherical equivalent refractive error; MLR = model based on multiple linear regression; REG = similar to MAG model, but the axial length and spherical equivalent refractive error were estimated by a convolutional neural network for regression.

∗ *P* < 0.0125.

† *P* < 0.05, compared to the average model.

**Figure 4:**
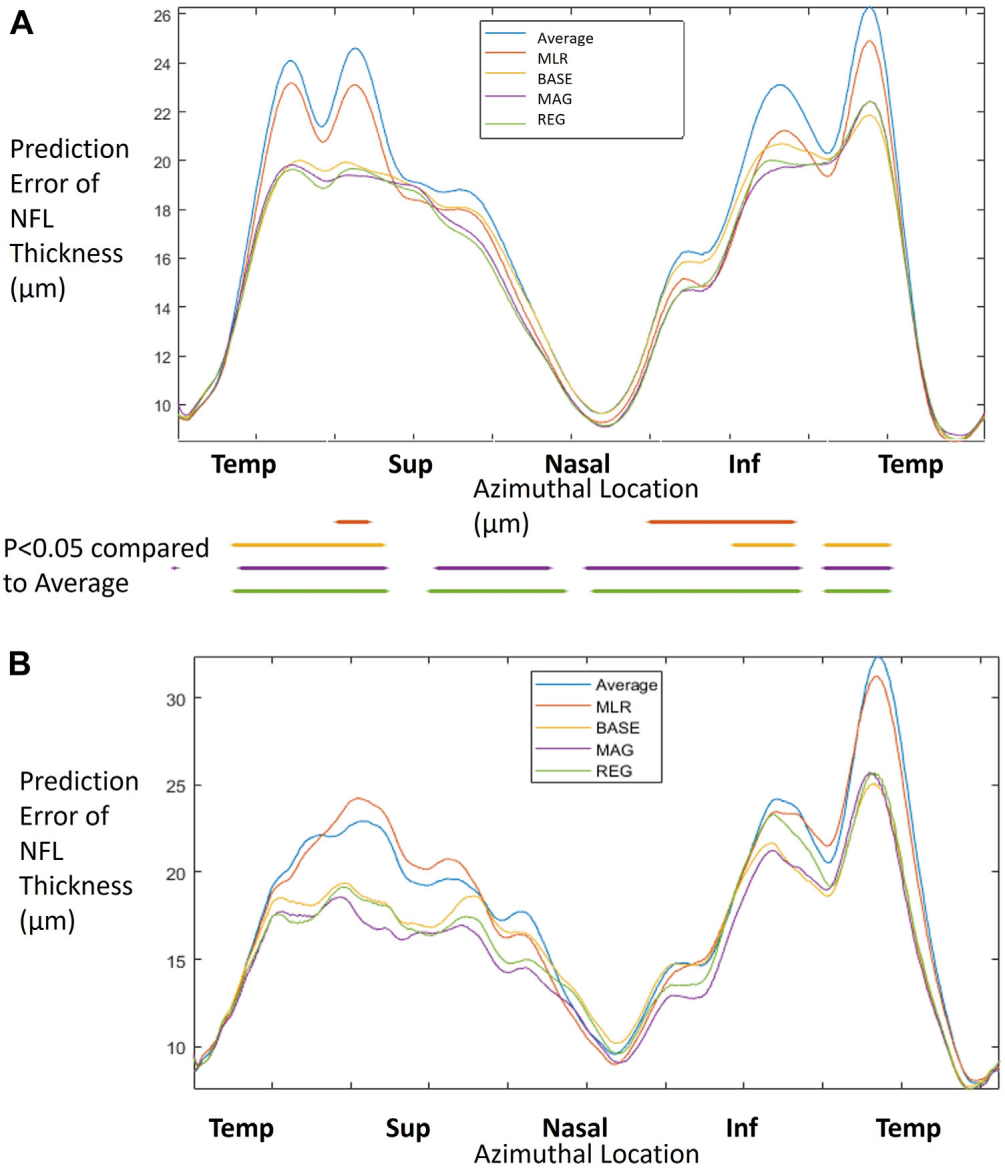
The prediction error profile (thin lines) and significance mask (*P* < 0.05 compared to the population average, thick lines below the axis). (**A**) Estimated from the healthy eyes from the Hong Kong Family cohort; (**B**) estimated from healthy eyes from the Casey Eye Institute cohort. In both figures, the prediction error is based on the root-mean-square of the profile difference (between true NFLT profiles and predicted normal reference or individualized baseline). Prediction error was summarized for the following 5 models: Average = population average without any adjustment; MLR = multiple linear regression model; BASE = deep learning models using only demographic information; MAG = BASE model plus magnification information; REG = BASE model with magnification estimated with a regression convolutional neural network. *P* value < 0.05 were used to check if there was a significant difference in prediction errors between the average and other models. NFL = nerve fiber layer; NFLT = nerve fiber layer thickness.

Based on the Hong Kong Dataset, the original (population average) model had large prediction errors near the 2 prominent NFLT peaks (Figure 4A). This was expected, as the location of these peaks (arcuate bundles) is known to vary between individuals. Compared with the original and MLR models, both the MAG and REG models greatly reduced the prediction error of the NFLT profile near the arcuate bundles. The BASE model also reduced the prediction error to a small degree. The MLR model did not significantly reduce the prediction error of NFLT at the arcuate bundles. This was expected, as the inputs to the MLR model did not contain information on the location of the arcuate bundles.

Similar trends were observed based on the CEI dataset (Table 3 and Figure 4B). All models showed lower prediction error for overall and quadrants than the population average. The MAG models had the smallest prediction error among other models, except in the inferior quadrant (Table 3). For the profile, the MAG DL model showed the smallest prediction errors in the superior quadrant and inferior-temporal sectors. All 3 DL models showed significantly smaller prediction error compared with both MLR and average models (root mean squares = 16.7 μm, 16.0 μm, and 16.6 μm vs. 18.7 μm and 18.8 μm, *P* < 0.001). The prediction errors from the CEI dataset were larger than those from the Hong Kong dataset for all parameters and all models, including the population average. This indicates a greater variability in the CEI dataset, possibly because it is multiracial. We did not analyze the effect of refractive error on the rate of false-positive abnormality in the CEI dataset because of the lack of highly myopic eyes.

### FPR

False positive rates were based on eyes with thickness below the fifth percentile cutoff of the normal reference. The cutoff was estimated from the histogram of the original NFLT or the adjusted NFLT in the emmetropia group. The REG model significantly reduced the FPR in the myopia and high myopia groups compared with the original value, except in the temporal area (Table 4). The REG model had a similar performance on FPR compared with the MLR or MAG model in most of the parameters (*P* > 0.05). However, the MLR and MAG models showed more consistent FPR in all groups. In hyperopia, all models had similar FPR (*P* > 0.05). For the temporal quadrant average of NFLT, the original value showed FPR significantly <5% in myopia groups, which might be due to the temporal NFL being less affected by magnification and the population variance in myopia groups being significantly lower than the emmetropia group.

**Table 4:** False Positive Rate Based on 5 Percentile Cutoff Estimated from Emmetropia Eyes (Hong Kong Family Cohort)

Eye Group	Models	Overall	Temporal	Superior	Nasal	Inferior
Hyperopia	Average	7.6%	5.1%	10.1%	3.2%	5.7%
	MLR	6.3%	4.4%	10.1%	5.7%	4.4%
	BASE	4.4%	5.1%	7.0%	4.4%	4.4%
	MAG	4.4%	5.7%	8.2%	5.7%	3.8%
	REG	5.1%	3.2%	10.1%	5.7%	5.1%
Low myopia	Average	13.3%	2.6%	13.9%	15.4%	10.1%
	MLR	8.3%∗	4.0%	8.7%∗	8.7%∗	6.3%∗
	BASE	13.5%†	3.8%	13.3%†	11.9%∗†	10.7%†
	MAG	6.7%∗	4.4%	7.1%∗	5.7%∗†	4.6%∗
	*REG*	9.1%∗	5.1%∗	7.7%∗	6.1%∗	6.1%∗
High myopia	Average	27.1%	3.1%	22.9%	31.3%	25.0%
	MLR	5.2%∗	7.3%	10.4%∗	6.3%∗	8.3%∗
	BASE	20.8%†	8.3%	20.8%†	16.7%∗†	18.8%
	MAG	6.3%∗	7.3%	4.2%∗	7.3%∗	6.3%∗
	REG	9.4%∗	4.2%∗	11.5%∗	5.2%∗	12.5%∗

BASE = deep learning model trained with conditions of vascular pattern and demographic information; MAG = BASE model with extra conditions of measured axial length and spherical equivalent refractive error; MLR = model based on multiple linear regression; REG = similar to MAG model, but the axial length and spherical equivalent refractive error were estimated by a convolutional neural network for regression.

∗ *P* value < 0.0125 compared to average.

† *P* value < 0.0125 compared to the MLR method.

### Difference between Normal Reference and 5 Percentile Cutoff

Due to the lack of verified glaucoma eyes in the Hong Kong dataset, we cannot evaluate the diagnostic sensitivity directly. Instead, we estimated the difference between each model's normal reference/baseline and the 5-percentile cut-point. A tighter (smaller) difference indicated that glaucomatous eyes at earlier stages with smaller loss of NFLT could be detected. For a population of glaucomatous eyes that includes those with early disease, the ability to detect smaller deviations from the healthy baseline would lead to better diagnostic sensitivity. Multiple linear regression and all DL models reduced the difference between the median and the 5-percentile cutoff compared with the population average (Table 5). Among 4 adjusting models, 2 DL models with magnification information had smaller differences than other models, and the BASE model had the worst performance, though those comparisons are not statistically significant (*P* > 0.0125).

**Table 5:** Difference between the Normal Reference Mean and 5 Percentile Cut-Point (Hong Kong Family Cohort)

Models	Overall	Quadrant Average			
		Temporal	Superior	Nasal	Inferior
Average	14.9 ± 0.7	14.5 ± 1.2	23.4 ± 1.8	18.9 ± 1.7	22.7 ± 0.9
MLR	13.7 ± 0.9	14.7 ± 1.4	21.8 ± 2.3	17.0 ± 2.2∗	20.5 ± 1.0†
BASE	14.2 ± 1.0	13.9 ± 1.3	21.0 ± 1.0†	17.3 ± 1.6∗	22.7 ± 0.9
MAG	13.3 ± 0.6†	13.7 ± 0.7	21.3 ± 0.7	15.6 ± 1.5∗	20.4 ± 1.0∗
REG	13.0 ± 0.6∗	14.1 ± 1.7	21.0 ± 0.6	15.2 ± 0.8∗	20.8 ± 1.6

BASE = deep learning model trained with conditions of vascular pattern and demographic information; MAG = BASE model with extra conditions of measured axial length and spherical equivalent refractive error; MLR = model based on multiple linear regression; REG = similar to MAG model, but the axial length and spherical equivalent refractive error were estimated by a convolutional neural network for regression.

∗ *P* < 0.0125.

† *P* < 0.05 compared to the population average model.

## Discussion

In this study, we proposed DL models to estimate the individualized baseline NFLT profile. By taking into account individual variations in the transverse optical magnification (related to axial eye length and refractive error) and anatomy (related to retinal vascular pattern), we hypothesize that the individualized baseline would help distinguish real pathology from normal interindividual variation and serve as a more reliable diagnostic reference than the simple population-average NFLT profile and sector averages. Our models are based on CVAE, which estimates the individualized baseline NFLT profile using the vascular pattern profile derived from the OCT scan, as well as demographic factors (age and gender).

In the classification of artificial intelligence (AI), CVAE is considered a type of probabilistic generative DL model, a category that also includes generative adversarial networks, diffusion models, and language models. The way that we use CVAE, however, is not probabilistic because the conditional input to the variational autoencoder is not random but is determined by patient characteristics such as vascular pattern, RPE elevation map, AL, and demographics. Thus, the baseline NFL profile predicted by our model is determined by the characteristics of the eye. Since we want to use the model to predict the baseline NFL profile that would have existed without disease damage, it is important that the model input is not affected by glaucoma or other diseases. This is an issue with the vascular pattern because glaucoma is known to attenuate retinal blood vessels.^46^ Thus, we inserted a step in the vascular pattern map generation to normalize the number of vessel pixels and prevent disease from affecting the overall vessel caliber.

We developed 3 DL models based on CVAE. The BASE model was only based on vascular patterns and demographics. The other 2 generative DL models also incorporated information on the transverse optical magnification based either on actual measurements of AL and SE (MAG) or an rCNN that estimated AL and SE using OCT-derived vascular pattern map and RPE elevation map (REG). We found that the vascular pattern helped to align the NFLT peaks of the individualized baseline to test eyes. We also found that individualized baseline models that incorporated actual or estimated magnification information significantly outperformed the population average in terms of the prediction error and reduced the false-positive glaucoma diagnosis rate in the myopic eyes. The BASE CVAE did not perform as well as MAG and REG, demonstrating that accounting for magnification-associated NFLT variation was still essential to the performance improvement achieved by our DL approach.

Given similar performance, the REG model may be preferable to MAG because it does not require additional AL and SE measurements. We found the 2 models to have similar prediction errors, but MAG was more effective in reducing the false-positive diagnosis rate in myopic eyes. The prediction error of the MAG model was smaller than that of the REG model in the validation with the CEI dataset. So, the REG model may need further improvement in generalizability. We believe that improvement may be possible by using wider maps of vascular pattern and RPE, as other investigators have found that wider-field disc photographs provided more accurate estimates of AL.^27^^,^^36^^,^^37^

Besides CVAE, other methods can also account for individual variation and reduce prediction error. Multiple linear regression was effective in reducing prediction error for overall and some quadrant NFLT,^19^^,^^47^, ^48^, ^49^ but not the NFLT profile. The ability of CVAE to reduce prediction error for NFLT profile in regions most susceptible to glaucoma damage (superior and inferior arcuate nerve fiber bundles) may be useful in improving the detection of focal glaucoma damage. The generative AI model may be better at predicting the NFLT profile because the location and bifurcation pattern of the NFL bundles may be correlated with the vascular pattern that serves as input to the CVAE. Other investigators have also used the retinal vascular pattern as input to machine learning algorithms to improve NFLT prediction.^13^^,^^15^^,^^16^^,^^50^^,^^51^ A potential advantage of our CVAE approach is that it provides highly individualized NFLT profile prediction. However, further studies are needed to compare the performance of the various approaches.

The results we presented showed that magnification-related information (AL and SE) and vascular pattern each significantly improved the accuracy of the individualized baseline reference generation. Either AL or SE alone also produced significant improvement (results not shown), but the combination was synergistic, and both clinical parameters are readily available. Therefore, we presented results for models using both AL and SE. While we did not present the individual evaluation of the importance of the other model inputs—age, sex, race, disc size, and signal strength—we found that each of them improved the performance of the models to small degrees. The effect of these predictive factors has already been shown in previously published studies.^6^^,^^15^^,^^19^^,^^33^^,^^47^^,^^52^, ^53^, ^54^, ^55^, ^56^, ^57^

There are several limitations to this study. First, most eyes in the Hong Kong dataset were East Asian, as the dataset was obtained from Hong Kong. Literature showed that East Asians had significantly or marginally thicker NFL than Whites and Blacks.^41^, ^42^, ^43^, ^44^, ^45^ We have tried a simple proportional adjustment for race as a postprocessing remedy, which works with reduced performance. It is possible that the local adjustment is different from the overall adjustment. Therefore, a better model might be achieved based on multiracial training data and using race as a condition. The second limitation is that the model was trained with a single OCT system—the Avanti. Therefore, retraining would be needed to apply this approach to other OCT systems. The third limitation of our study is that the diagnostic sensitivity has not been tested on a group of glaucoma patients. As a more specific normative reference, we believe that the individualized baseline will improve the accuracy of NFL focal loss analysis. Our next step will be to develop an algorithm to detect focal NFL loss and assess its performance in glaucoma diagnosis. A fourth limitation is that deviation from normal reference does not necessarily indicate the presence of glaucoma—it could be due to other ocular diseases. Therefore, our individualized baseline would also need to be assessed in patients with other ocular comorbidities.

A potential pitfall of our approach is that eye diseases could affect the retinal vascular pattern and thereby the generation of the individualized NFLT baseline reference. Both glaucoma and diabetic retinopathy are known to cause attenuation of retinal vascular caliber, which may cause the AI model to generate attenuated NFLT and reduce our ability to detect NFLT loss. To make our model more disease invariant, we normalized the vessel size to keep a fixed ratio of vascular to nonvascular pixels in the vascular pattern profiles. The effectiveness of this approach will be tested in future studies on glaucoma diagnostic accuracy.

A similar pitfall is that glaucoma could affect the ILM elevation map, leading to errors in the estimation of magnification-related factors AL and SE. For that reason, we used the RPE elevation map, which would not be affected by glaucomatous thinning of NFLT, to estimate AL and SE in the MAG model.

We have demonstrated that the generative DL approach can generate individualized NFLT profiles, sectors, and overall values that reduce prediction error relative to simple population averages. This approach can be extended to other OCT metrics and OCT angiography metrics, such as macular ganglion cell complex thickness, NFL plexus capillary density, or cup-disc ratio.

### Conclusions

We developed a generative DL AI model that can provide an individualized NFLT baseline using vascular patterns from OCT only. Compared with models based on multiple regression, the individualized baseline performed equally in reducing the population variance of global NFLT in healthy eyes or FPR in detected NFLT abnormality in myopia but performed better in reducing the variance locally. The approach to constructing the individualized baseline could be extended to other OCT and OCT angiography metrics.

## Declaration of Generative AI and AI-Assisted Technologies in the Writing Process

During the preparation of this work, the authors used Grammarly to correct the grammar. After using this tool/service, the authors reviewed and edited the content as needed and take full responsibility for the content of the publication.
